# Genomics of autism spectrum disorder: approach to therapy

**DOI:** 10.12688/f1000research.13865.1

**Published:** 2018-05-22

**Authors:** Fatma Ayhan, Genevieve Konopka

**Affiliations:** 1 Department of Neuroscience, UT Southwestern Medical Center, Dallas, 75390-9111 TX, USA

**Keywords:** autism, genomics, genetics, iPSCs, organoids, single-cell RNA-sequencing

## Abstract

Autism spectrum disorder (ASD) is a highly prevalent neurodevelopmental condition with no current treatment available. Although advances in genetics and genomics have identified hundreds of genes associated with ASD, very little is known about the pathophysiology of ASD and the functional contribution of specific genes to ASD phenotypes. Improved understanding of the biological function of ASD-associated genes and how this heterogeneous group of genetic variants leads to the disease is needed in order to develop therapeutic strategies. Here, we review the current state of ASD research related to gene discovery and examples of emerging molecular mechanisms (protein translation and alternative splicing). In addition, we discuss how patient-derived three-dimensional brain organoids might provide an opportunity to model specific genetic variants in order to define molecular and cellular defects that could be amenable for developing and screening personalized therapies related to ASD.

## Introduction

Autism spectrum disorder (ASD) is a phenotypically and genetically heterogeneous neurodevelopmental condition that manifests as deficits in reciprocal social interaction, repetitive behavior patterns, and restricted interests ^1^. The prevalence of ASD is as high as 1 in 68 children ^2^ in the US, and ASD has a profound impact at the individual, family, and societal levels. Although environmental factors likely play some role in the etiology of ASD ^3^, family and twin studies show that genetics contribute to the majority of the risk associated with ASD ^4– 9^. Genome-wide studies using genotyping microarrays, whole exome sequencing (WES), and whole genome sequencing have identified a rapidly growing number of genes linked to ASD ^10– 21^, providing a window into the molecular underpinnings of the disorder. However, our understanding of molecular mechanisms anchored to this heterogeneous group of genetic variants is not entirely clear. The paucity of disease-modifying therapies or molecular diagnostic tools for ASD makes identifying molecular disease mechanisms critical to assist developing rationally designed therapies. Additionally, details regarding the time course of molecular alterations in ASD can inform diagnostic biomarkers and quantitative measures to indicate disease severity and evaluate the efficacy of future therapeutic approaches.

Here, we review the recent progress in understanding the underlying genetics of ASD, including the identification of inherited, *de novo*, and somatic mutations linked to the disease. We then discuss how convergent disease mechanisms in ASD can potentially translate into the most appropriate biomarker development and treatment strategies for individuals or subtypes (or both) with ASD. Finally, we consider the unprecedented premise of patient-derived three-dimensional (3D) brain organoids as appropriate models to test and validate the functional impact of identified genetic variants as accessible and flexible platforms to screen and test for therapeutic agents.

## The complex genetic makeup of autism spectrum disorder

The importance of heritable genetic variability in ASD pathogenesis has been highlighted in twin and family studies. The increased prevalence of the disease in siblings of ASD patients and greater ASD concordance rates in monozygotic twins compared with dizygotic twins has prompted significant efforts toward understanding the genetic architecture of ASD pathophysiology. Although the identification of mutations linked to monogenic syndromic forms of ASD, including Fragile X ^22^, Rett ^23^, *MECP2* duplication ^24^, tuberous sclerosis complex ^25^, *PTEN* macrocephaly ^26^, and Timothy ^27^ syndromes, provided key insights into the genetic basis of ASD, these rare syndromes collectively account for only about 5% of ASD cases ^28^, leaving the etiology of non-syndromic ASD cases mostly unknown. The highly heterogeneous disease presentation of non-syndromic ASD initially posed serious impediments for identifying reproducible ASD-associated mutations. Despite these challenges, the assembly of large patient cohorts along with advances in genomic technologies within the last decade has facilitated the identification of ASD-associated variants in hundreds of genes, including single-nucleotide variant (SNVs) and copy number variants (CNVs) ^29^. The use of WES and whole genome sequencing in family cohorts with sporadic ASD (simplex) and with more than one affected individual (multiplex) led to the discovery of both rare inherited and *de novo* ASD risk variants. Rare recessive mutations have been reported in genes such as *CNTNAP2* ^30^, *SLC9A9* ^31^, *AMT* ^20^, *PEX7* ^20^, *CC2D1A* ^32^, and *BCKDK* ^33^ in consanguineous families with ASD and epilepsy, highlighting the role of recessive inheritance of deleterious mutations associated with ASD. The role of inherited variants in ASD was further supported through WES in larger cohorts of unrelated families ^34, 35^. WES in large cohorts of simplex families (one affected child sequenced together with unaffected parents) provided substantial insight into the role of *de novo* (or spontaneous) genetic variants in ASD. Numerous studies have reported increased rates of rare *de novo* CNVs and SNVs in individuals with ASD ^10, 16– 18, 36^ and have identified high-confidence ASD genes, including *CHD8* ^16, 18, 37– 39^, *SYNGAP1* ^21, 40, 41^, *DYRK1A* ^42^, and *SCN2A* ^16^. Moreover, targeted sequencing approaches confirmed the recurrence of some of these *de novo* mutations in independent cohorts, substantiating their role in ASD pathogenesis ^10, 36, 39^. Finally, one very interesting group of genetic variants that has recently been implicated in ASD is somatic mutations ^43– 45^. Somatic mutations can occur during development and yield mosaic individuals with distinct cellular genomes in subsets of their somatic cells ^46^. Whereas routine genetic sampling from blood misses the disease-associated somatic variants in the brain, targeted sequencing on ASD post-mortem tissue has detected increased rates of deleterious somatic mutations in cases compared with controls ^45^. Interestingly, there may be some overlap of genes at risk for both germline and *de novo* somatic mutations (for example, *SCN2A*) ^43^. Future single-cell sequencing approaches ^47^ will be informative to identify and characterize cells that carry disease-related somatic mutations ^48^.

Taken together, recent advances in ASD gene discovery highlight the complexity of the genetic landscape of the disease while beginning to shed light on some of the biological pathways at risk in ASD. This complexity is underscored by the potential for certain combinations of common genetic variants contributing to ASD by increasing an individual’s susceptibility to pathogenic effects of rare inherited, *de novo*, or somatic mutations. Given the progress in identifying high-confidence risk genes for ASD, investigators can now direct their attention to understanding the pathogenicity of this genetic variance and identifying potential common convergent disease mechanisms as molecular targets for future treatment strategies.

## Convergent molecular mechanisms

One approach to understand pathogenesis and identify therapeutic targets amid a complex genetic architecture is to elucidate downstream pathways commonly affected across ASD cases with distinct genetic etiologies. One example of a convergent molecular mechanism includes defects in the regulation of protein synthesis and alternative splicing (AS) as potential unifying pathways for ASD ^49^.

Precise regulation of translation at synapses during the tight window of a learning experience has been shown to be extremely critical for the formation and maintenance of long-term memory ^50^. Several mutations in translation factors and regulators such as *eIF4E* ^51, 52^, *TSC1/2* ^53^, and *PTEN* ^54^ are associated with ASD, underscoring the involvement of translational defects in ASD pathogenesis. Furthermore, there is emerging evidence showing dysregulated translational activity in cells derived from non-syndromic ASD patients, including aberrant activity of mammalian target of rapamycin (mTOR), a key regulator of translation, suggesting translational dysregulation as a shared pathogenic mechanism in genetically distinct ASD cases ^55, 56^. The inhibition of aberrant translation directly via compounds targeting translation factors (for example, 4EGI-1 ^57^) or by modulating the mTOR pathway ^58^ has been shown to prevent autism-relevant phenotypes in mice and has been proposed as a therapeutic strategy to correct dysregulated protein synthesis in ASD ^58^.

AS is co- or post-transcriptionally regulated by RNA-binding proteins (RBPs) and tightly controlled during developmental stages in a tissue-specific manner ^59^. Given the limited number of protein-coding genes in the human genome, AS is recognized as an essential source of transcriptomic and proteomic diversity driving the species-specific features of the human brain ^60– 62^. Dysregulation of AS in post-mortem brain tissue from ASD patients with distinct etiologies has been increasingly apparent as a convergent mechanism in ASD ^63– 65^. The transcripts that are misspliced in ASD are enriched for neuronal RBP targets, including those of RBFOX1 ^63, 65^, SRRM4 ^65^, and PTBP1 ^65^, suggesting that defective RBP function is a common feature of ASD. Genetic evidence showing ASD-linked chromosomal translocations and copy number variations in *RBFOX1* also supports a prominent role for loss or dysregulation (or both) of RBFOX1 activity in ASD pathogenesis ^13, 66– 68^. Loss of RBFOX1 in mice causes deficits in synaptic transmission ^69^ and corticogenesis ^70^. Neuronal-specific, activity-dependent, 3- to 27-nucleotide microexons are frequently misspliced in ASD ^64, 71^. This group of genes that are subject to microexon splicing is enriched for synaptic functions and ASD genes ^64, 71^. These microexons are regulated primarily by the neuronal RBP, SRRM4, which is downregulated in ASD brains ^64^. Haploinsufficiency of SRRM4 in mice resulted in microexon misregulation and ASD-like features, including altered social behaviors ^71^. These data highlight the function of RBPs, including RBFOX1 and SRRM4, as essential for cortical development and function and at risk in ASD. Taken together, global dysregulation of RNA processing and protein translation is likely to be a common feature of genetically diverse ASD cases, and the regulation of these processes might be a viable target for therapeutic approaches.

## Patient-specific disease models

The high degree of genetic heterogeneity in ASD requires personalized approaches to understand the underlying individual pathogenic mechanisms and develop efficient treatments. In addition, there is a need for improved model systems with appropriate genetic backgrounds to test identified convergent biological mechanisms such as the ones discussed above. Advances in stem cell biology in the last decade have yielded protocols for the generation of human neurons from accessible somatic tissue (for example, skin), ** overcoming the unavailability of human neurons from specific developmental stages or disease states. Briefly, human induced pluripotent stem cells (hiPSCs) are generated by the ectopic expression of specific transcription factors in somatic cells that then can be differentiated into neurons or glia harboring the genetic features of the human individual from whom the cells are derived, either the patients or matched unaffected controls ^72^. In addition, isogenic neurons generated by introducing mutations in control iPSCs via gene editing technologies—that is, CRISPR-Cas9 ^73, 74^ and TALENs ^75^—can be used to study the functional impact of disease-related mutations on a non-disease genetic background.

Research adopting iPSC-based models has begun to impact the understanding of the biological underpinnings of several ASD-related genetic variants ^76, 77^. In several instances, syndromic forms of ASD, including Fragile X ^78, 79^, Rett ^80^, Timothy ^81, 82^, and Phelan–McDermid ^83^ syndromes, have been modeled by using iPSCs. These studies have defined disease-related defects in patient-derived neurons, including reduced synaptic density, impaired excitatory transmission, and aberrant signaling. Additionally, a recent study of iPSCs from an ASD patient with a *de novo* mutation in *TRPC6* confirmed the potential for patient-specific disease modeling of rare ASD variants ^84^.

Breakthroughs in iPSC culture systems have facilitated the generation of more complex differentiation programs that yield organ-like structures. These 3D brain organoids have been established with the goal of improved recapitulation of brain development and connectivity *in vitro*, providing an unprecedented opportunity to study human brain features in a dish ^85– 87^. A major goal of using patient-derived 3D organoids is to perform high-throughput drug screens to correct ASD-relevant cellular defects and reliably predict drug responses specific to each individual. In the future, standardization of 3D human brain organoid generation is needed for reliable and reproducible disease modeling. Defining the functional properties and molecular signatures of brain organoids derived from unaffected iPSCs at several time points will provide insights into how this model system follows *in vivo* human brain development and baseline information for disease modeling. It will be important to address how the differentiation process of 3D brain organoids corresponds to stages of human brain development. This will be essential to identify and translate the critical time window for successful therapeutic intervention. Recent advances in single-cell RNA sequencing facilitate the identification of cell types and differentiation states of diverse human neuronal populations in fetal brain *in vivo* ^88^ and have also proven to be very useful for characterizing brain organoids ^89^. Integration of cell-specific gene expression profiles with regional and developmental timing mechanisms has been elegantly carried out from human fetal tissue ^88^, and these data can be superimposed on the data derived from patient organoids to identify aberrant profiles. Inherent limitations of 3D brain organoids such as lack of behavioral output and circuit-based studies should be addressed with complementary studies using animal models ^19^.

In terms of cell-specific profiling, most research has gone into characterizing the neuronal defects in ASD; however, the involvement of glia has recently been implicated in many neuropsychiatric diseases ^90^. For example, a recent iPSC model provided evidence that defects in astrocytes can contribute to non-syndromic ASD ^91^ with unknown genetic cause. Therefore, future strategies to develop therapies for ASD should not only focus on neurons but also include all cell types in the brain. In addition, these data support the promise of using iPSC models from individuals with genetically complex etiologies to narrow the therapeutic search window to common pathogenic mechanisms. Improvements to brain organoid models that include many cell types such as glia and endothelial cells from non-syndromic ASD patients should further facilitate the identification of patient-specific cellular deficits.

## Conclusions

Technological and conceptual advances in genomics, stem cell biology, and gene editing together with large cohorts of patients are providing opportunities to identify genetic causes of ASD and develop functionally relevant disease models. Integrative studies that include post-mortem tissue, genomics, and single-cell transcriptomics will continue to provide insights into human brain development and how this process is disrupted in ASD. By improved modeling of the disease using patient tissues and incorporating data from genomic and gene expression studies into these models, the field should move closer to developing personalized therapeutic approaches as well as identifying common druggable molecular pathways. Thus, persistent pursuit of all of the strategies discussed above will be needed to define optimal personalized treatments that potentially could involve several drugs in combination for additive or synergistic effects.

## Abbreviations

3D, three-dimensional; AS, alternative splicing; ASD, autism spectrum disorder; CNV, copy number variant; hiPSC, human induced pluripotent stem cell; mTOR, mammalian target of rapamycin; RBP, RNA-binding protein; SNV, single-nucleotide variant; WES, whole exome sequencing.
